# Gastric Mucormycosis: An Infection of Fungal Invasion into the Gastric Mucosa in Immunocompromised Patients

**DOI:** 10.1155/2020/8876125

**Published:** 2020-09-16

**Authors:** Haider A. Naqvi, Muhammad Nadeem Yousaf, Fizah S. Chaudhary, Lawrence Mills

**Affiliations:** ^1^Department of Medicine, Medstar Union Memorial Hospital, Baltimore, MD 21218, USA; ^2^Department of Medicine, MedStar Good Samaritan Hospital, Baltimore, MD 21239, USA; ^3^Department of Medicine, MedStar Franklin Square Medical Center, Baltimore, MD 21237, USA; ^4^Department of Medicine, MedStar Harbor Hospital, Baltimore, MD 21225, USA; ^5^Department of Gastroenterology, MedStar Good Samaritan Hospital, Baltimore, MD 21239, USA

## Abstract

Primary gastric mucormycosis is a rare but potentially lethal fungal infection due to the invasion of Mucorales into the gastric mucosa. It may result in high mortality due to increased risk of complications in immunocompromised patients. Common predisposing risk factors to develop gastric mucormycosis are prolonged uncontrolled diabetes mellitus with or without diabetic ketoacidosis (DKA), solid organ or stem cell transplantation, underlying hematologic malignancy, and major trauma. Abdominal pain, hematemesis, and melena are common presenting symptoms. The diagnosis of gastric mucormycosis can be overlooked due to the rarity of the disease. A high index of suspicion is required for early diagnosis and management of the disease, particularly in immunocompromised patients. Radiological imaging findings are nonspecific to establish the diagnosis, and gastric biopsy is essential for histological confirmation of mucormycosis. Prompt treatment with antifungal therapy is the mainstay of treatment with surgical resection reserved in cases of extensive disease burden or clinical deterioration. We presented a case of acute gastric mucormycosis involving the body of stomach in a patient with poorly controlled diabetes and chronic renal disease, admitted with acute onset of abdominal pain. Complete resolution of lesion was noted with 16 weeks of medical treatment with intravenous amphotericin B and posaconazole.

## 1. Introduction

Gastric mucormycosis is a rare but lethal fungal infection due to invasion of Mucorales (a filamentous fungus) into the gastric mucosa that may result in high mortality (up to 54%) in immunocompromised patients [1]. An estimated 75% of mucormycosis (formally known as zygomycosis) infection is caused by the fungi class of zygomycetes and particularly Mucor, Rhizopus, or Rhizomucor species [1–3]. Common sites of mucormycosis are upper respiratory tract, nasal or paranasal sinuses, skin, orbit, and brain; however, the gastrointestinal tract is rarely infected [4]. Serologic biomarkers are nonspecific; however, a biopsy from the affected site is the gold standard to establish histologic diagnosis of mucormycosis. A positive culture or polymerase chain reaction (PCR) on biopsy specimen is occasionally required for diagnosis confirmation. Medical management with antifungal therapy such as lipid formulation of amphotericin B, posaconazole, and newer agents isavuconazole or triazole is the mainstay option for treating gastric mucormycosis [5, 6]. For patients with severe disease such as tissue necrosis and those with late presentation, a combination of both medical management with early surgical resection may improve the outcome of disease. We present a case of an immunocompetent individual who developed gastric mucormycosis.

## 2. Case Presentation

**Figure 1:**
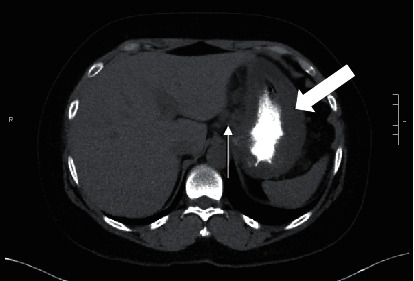
CT scan of the abdomen displaying the transmural thickening of the stomach (white blocked arrow) and the perigastric inflammation with reactive adenopathy (white arrow).

**Figure 2:**
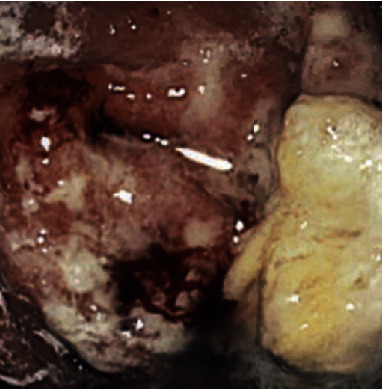
Upper endoscopy showing ulcerated lesion in the gastric fundus with the overlaying bleeding and exudative material.

**Figure 3:**
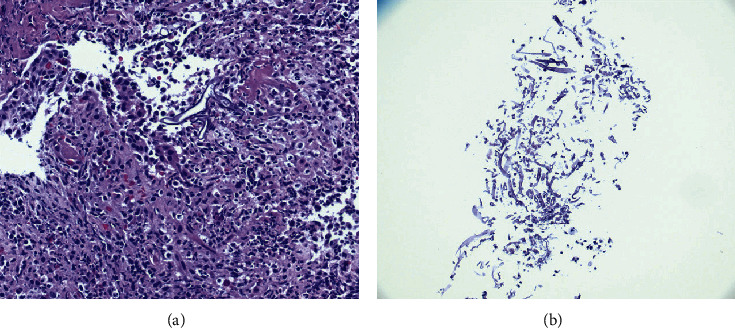
Histological examination of lesion showing polymorphonuclear neutrophils indicating inflammation of gastric mucosa (a) and fragments of invading fungal hyphae (b).

**Figure 4:**
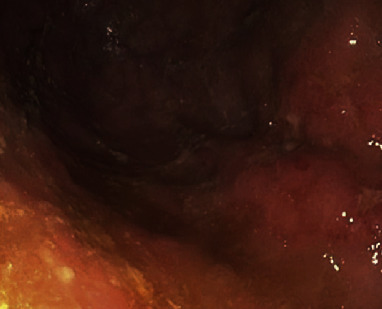
Repeat EGD showing cleared infection and ulceration in the fundus of the stomach.

A 55-year-old female with past medical history of insulin-dependent uncontrolled diabetes mellitus and stage IIIb chronic kidney disease presented to the emergency room with acute abdominal pain for two weeks. The abdominal pain was epigastric, 7/10 in severity, and nonradiating, without precipitating or relieving factors. It was associated with nausea and multiple episodes of nonbiliary, nonbloody emesis. On physical examination, vitals were unremarkable. Epigastric tenderness was noted on abdominal examination. Laboratory workup was remarkable for leukocytosis (11.2 k/ul), elevated lipase (589 u/L), and elevated creatinine (1.62 mg/dL). Computed tomography (CT) of the abdomen and pelvis (Figure 1) demonstrated transmural thickening at gastric body and fundus, with associated perigastric inflammation and reactive adenopathy. Esophagogastroduodenoscopy (EGD) revealed multiple ulcerated sessile masses in the gastric fundus with a large exudate covering mass (Figure 2). Biopsy of mass revealed polymorphonuclear neutrophils indicating inflammation of gastric mucosa and fragments of invasive fungal hyphae (zygomycetes) with polymerase chain reaction (PCR) and culture of the biopsy specimens detecting *Rhizopus microsporus* DNA (Figures 3(a) and 3(b)). The patient was not amenable to the surgical resection because of multiple comorbidities and high risk for surgical complications. She was medically treated with intravenous amphotericin B 5 mg/kg daily that was later switched to posaconazole 300 mg daily due to worsening renal function and poor tolerance. Surveillance of the gastric lesions was performed with serial EGDs. Complete resolution of gastric mucormycosis with the absence of hyphae was noted on endoscopic gastric biopsies after 16 weeks of antifungal therapy (Figure 4).

## 3. Discussion

An estimated prevalence of mucormycosis is 0.16 (0.12 to 0.20) per 10,000 patients [7]. The prevalence rate of mucormycosis amongst patients with uncontrolled diabetes is 36% [8]. We presented a case of acute gastric mucormycosis in a patient with poorly controlled diabetes and chronic renal disease, admitted with acute onset of abdominal pain. An uncontrolled diabetes and CKD stage IIIb were major risk factors predisposing to the development of gastric mucormycosis. The patient's symptoms were not amenable to proton pump inhibitors which prompted further investigation including abdominal radiological imaging and upper endoscopy. Radiological imaging findings were nonspecific; however, EGD revealed multiple ulcerated sessile masses located at greater curvature and gastric fundus. These mucosal lesions were covered with a large grayish exudate, highly suspicious for gastric mucormycosis. The diagnosis of gastric mucormycosis was confirmed with histological examination, culture, and PCR of biopsy specimen.

Gastrointestinal mucosal involvement could be the sole manifestations of mucormycosis that accounts for approximately 7% of all reported cases [1]. Among gastrointestinal mucormycosis, the stomach is the most commonly affected organ (67%), followed by the colon (21%), small intestine (4%), and esophagus (2%) [1, 4, 8, 9]. The invasion of Mucorales into the body occurs through inhalation, ingestion, or inoculation of the spores [8]. The ingestion of the spores possibly from being present in fermented milk, dried bread products, fermented porridges, and alcoholic beverages derived from infected corn is the primary mode of Mucorales entry into the gastrointestinal tract. And even possibly from infected tongue depressors at physician clinics [2]. An iatrogenic gastric mucormycosis has been reported in a study from using a wooden tongue depressor and wooden applicators for crushing and mixing medication for a critically ill patient on tube feeding [10]. While immunocompetent hosts can fight off the invasion of Mucorales after ingestion of the spores into the alimentary tract, immunocompromised individuals are not able to resist mucosal invasion due to poor defence mechanism and are prone to develop severe infection. The pathogenesis of infection in diabetic patients with or without diabetic ketoacidosis (DKA) remains to be elusive at this time. The proposed mechanism of gastric mucormycosis infection is the phagocytic dysfunction, impaired chemotaxis, and defective intracellular destruction of Mucorales in the presence of acidic environment of the stomach [11]. Uncontrolled diabetes and CKD may produce slightly acidic environment in the body similar to a diabetic ketoacidosis and predispose patients to mucormycosis.

In the current literature, multiple case reports on gastric mucormycosis have shown abdominal pain as the most common presenting symptom followed by hematemesis, melena, hematochezia, nausea, vomiting, dysphagia, and odynophagia (Table 1) [4, 9, 12–44]. The predisposing risk factors to develop gastric mucormycosis are prolonged uncontrolled diabetes mellitus with or without diabetic ketoacidosis (DKA), solid organ or stem cell transplantation, underlying hematologic malignancy, major trauma, utility of steroids, disseminated chronic infections, iron overload states, and severe neutropenia (Table 1) [4, 9, 12–44]. The gastric body is the most commonly affected location of gastric mucormycosis. The invasion of blood vessels under the mucosal surface may result in life-threatening gastrointestinal hemorrhage and is a poor prognostic factor of disease [4].

The radiological modalities such as CT scan or MRI of the abdomen usually reveal nonspecific findings such as mucosal wall thickening, mass, and reactive lymphadenopathy and prompts additional investigation with endoscopic or surgical biopsy of the lesions. EGD finding of ulcerated patchy mucosal lesions with overlying greenish or greyish exudate is a characteristic feature of gastric mucormycosis; however, biopsy of lesions is essential to differentiate it from gastric malignancy [4, 8, 21]. EGD alone does not help to make a definitive diagnosis, and additional testing with biopsy specimens is required. Histopathological and culture testing is the most definite in establishing the diagnosis. Direct microscopy, using optical brighteners, and hematoxylin and eosin stains have shown rapid visualization of the morphological structure of fungi for diagnosis of mucormycosis [45]. In addition, neutrophilic infiltration can also be seen on histological examination. PCR and fungal cultures of the specimen are occasionally needed for absolute confirmation of diagnosis. The cultures are performed on the Sabouraud agar, and rapid growth of fungi can be seen within three to seven days. Molecular-based assays, such as PCR, have shown sensitivity and specificity closer to 100% in comparison to microscopic and histopathological assays and have been noted to yield faster test results [45].

Early initiation of antifungal therapy within 6 days was found to have better patient outcomes and lower mortality rates [9, 46]. Multiple studies have shown the medical management with antifungal therapy is the mainstay treatment; however, a combination of antifungal with surgical management with either debridement or gastrectomy of mucormycosis lesions may be required in a significant number of cases specifically in those with severe disease and multiple comorbidities (Table 1) [4, 9, 12–44]. In vitro studies have shown the resistance of Mucorales to many antifungal medications such as fluconazole, ketoconazole, voriconazole, itraconazole, flucytosine, and echinocandins [47]. Commonly used antifungal medications are lipid formulation of amphotericin B (45% to 52%) and posaconazole (45%) in hospitalized patients with mucormycosis [7]. The dosage of amphotericin B remained under debate for a long time, and decision to use low versus high dose should be based upon individual patient factors including drug tolerability, side effects, and disease response to medical therapy. Currently, a dose of 5 mg/kg body is recommended by majority of physicians with a gradual escalation of dose up to 10 mg/kg for effective control of disease [45, 48, 49]. Among patients with poor tolerability of amphotericin B and those with the risk of potential side effects such as nephrotoxicity, an alternative antifungal, posaconazole, is recommended. Posaconazole is an oral agent which can also be used as maintenance agent and for long-term prophylaxis in the management of gastric mucormycosis. Although it is better tolerated than amphotericin B, in patients with hematological malignancy and gastric mucormycosis, erratic absorption of drug is concerning which may result in breakthrough infection due to suboptimal concentration of serum posaconazole. In our case, the patient was initially started on first-line antifungal agent liposomal amphotericin B. It was stopped in two weeks due to development of worsening renal function and poor tolerability. It was switched with posaconazole 300 mg daily that was also used as maintenance and salvage therapy. Posaconazole was continued for roughly four months thereafter when repeat EGD showed complete resolution of gastric mucormycosis. The duration of antifungal therapy at this time appears to be controversial given insufficient data. Serial EGD surveillance of gastric lesions is required to determine the effect of the therapy. Newer antifungal agents isavuconazole and triazole are also being utilized; however, their efficacy in resolution of the disease is still under investigation.

At times, management is not only sufficient with antifungal therapy alone and certain patients require surgical intervention. A combination of antifungal and surgical resection of lesions is indicated in case of extensive disease, angioinvasion, mucosal necrosis, and those with failed medical therapy [26, 45]. Aggressive surgical resection of necrotic lesions and negative tissue margins of fungal invasion may prevent dissemination of disease and its complications such as bowel perforation, peritonitis, and massive hemorrhage [2].

## 4. Conclusion

Mucormycosis is a fatal opportunistic fungal infection which may result in high mortality in untreated patients. A high index of suspicion and awareness of physicians is required for early diagnosis and management of disease particularly in immunocompromised patients. A typical presentation such as worsening abdominal pain should be investigated with CT scan or MRI and EGD in those with inconclusive finding on radiological imaging. Biopsy of the suspected mucosal lesions is the diagnostic of gastric mucormycosis. Immediate medical management with antifungal agents such as liposomal amphotericin B or posaconazole is required given the invasive nature of disease and high mortality. Serial EGD may be essential to monitor healing of gastric mucormycosis and determine duration of medical management. A combination of medical and surgical resection of mucosal lesions is warranted in the case of poor response to antifungal therapy and those with extensive disease burden.

## Figures and Tables

**Table 1:** Clinical presentation and management of gastric mucormycosis.

Study	Year	Age	Gender	Presenting symptoms	Predisposing factor	Location	Treatment	Outcome
Sharma [12]	2020	66	F	Abdominal pain	N/A	Body	Total gastrectomy and amphotericin B	Alive
Madireddy [13]	2020	28	M	Melena	Diabetes mellitus	Fundus	Gastrectomy, amphotericin B, and posacanozole	Alive
Buckholz and Kaplan [14]	2020	63	M	Epigastric pain	Hematological malignancy	Body and fundus	Total gastrectomy and amphotericin B	Died
Platt et al. [15]	2019	33	F	Epigastric pain, nausea, and vomiting	Immunosuppressive medication	Cardia	Amphotericin B and surgical debridement	Died
Yusuf et al. [16]	2019	51	M	Hematemesis	Hematological malignancy	Cardia	Amphotericin B	N/A
Adhikari et al. [17]	2019	57	F	Hematemesis	N/A	N/A	Amphotericin B and posacanozole	Alive
Uchida et al. [4]	2019	82	F	Epigastric pain and melena	Immunosuppressive medication	Upper body and fundus	Amphotericin B	Died
Guddati et al. [18]	2019	42	M	Hematemesis	N/A	Multiple sites	Total gastrectomy	Alive
Sehmbey et al. [19]	2019	48	M	Coffee ground emesis	Trauma	Body and fundus	Amphotericin B	Died
Alfano et al. [20]	2018	42	F	Abdominal pain and melena	Organ transplantation	Posterior wall	Amphotericin B and posacanozole	Alive
Termos et al. [9]	2018	52	F	Abdominal pain	Diabetes	N/A	Amphotericin B and total gastrectomy	Died
Abreu et al. [50]	2018	23	F	Diffuse abdominal pain and vomiting	N/A	N/A	Total gastrectomy and amphotericin B	Alive
Gani et al. [21]	2018	79	M	Dysphagia and odynophagia	Organ transplantation	N/A	Isavuconazole	Alive
Sánchez-Velázquez et al. [22]	2017	53	F	Hematemesis	N/A	Cardia	Total gastrectomy	Died
Kgomo et al. [23]	2017	38	F	Hematemesis	HIV	Entire stomach	Amphotericin B	Died
Chow et al. [24]	2017	34	M	Abdominal pain and fever	Trauma	Fundus and cardia	Subtotal gastrectomy	Died
Nasa et al. [25]	2017	31	M	Abdominal distention	N/A	N/A	Amphotericin B	Died
Lee and Lee [26]	2016	41	F	Melena	Trauma	N/A	Amphotericin B	Died
Kim et al. [27]	2016	45	M	Weakness	Diabetic ketoacidosis	Entire stomach	Amphotericin B and gastrectomy	Alive
El Hachem et al. [28]	2016	67	M	Dysphagia and epigastric pain	N/A	Greater curvature	Amphotericin B and posacanozole	Alive
Sethi et al. [29]	2016	58	M	Hematemesis	Organ transplantation	Antrum and distal body	Amphotericin B	Alive
Putrus et al. [30]	2015	65	M	Abdominal pain	Diabetes mellitus	Cardia and body	Amphotericin B and total gastrectomy	Died
Alvarado-Lezama et al. [31]	2015	32	M	Abdominal pain	Diabetic ketoacidosis	N/A	Total gastrectomy	Died
Raviraj et al. [32]	2015	19	F	Fever, vomiting, and diarrhea	N/A	N/A	Amphotericin B and posacanozole	Alive
Kulkarni and Thakur [33]	2014	50	M	Abdominal pain and distention	Diabetes	Gastric body	Surgical intervention	Died
Lee et al. [34]	2014	55	M	Severe abdominal pain	N/A	N/A	Subtotal gastrectomy and amphotericin B	Alive
Katta et al. [35]	2013	60	M	Unconsciousness	N/A	Cardia and fundus	Amphotericin B and posacanozole	Alive
Dutta et al. [36]	2012	64	F	Abdominal distention and odynophagia	N/A	Fundus and body	Amphotericin B	Alive
Chhaya et al. [37]	2011	53	F	Melena	N/A	Body	Amphotericin B	Alive
Berne et al. [38]	2009	55	M	Postoperative fever	N/A	Greater curvature	Total gastrectomy and amphotericin B	Died
Prasad and Nataraj [39]	2008	28	M	Abdominal pain, bloody diarrhea, and fever	N/A	Lesser curvature	Amphotericin B	Died
Ho et al. [40]	2007	58	W	Hematemesis	N/A	Posterior wall	Amphotericin B	Alive
Paulo De Oliveria and Milech [41]	2002	17	F	Epigastric pain	Diabetic ketoacidosis	Greater curvature and posterior wall	Amphotericin B	Died
Sheu et al. [42]	1998	70	M	Fever, epigastric pain, and melena	Organ transplantation	Greater curvature	Amphotericin B	Died
Winkler et al. [43]	1996	37	F	Melena	Organ transplantation	N/A	Amphotericin B	Alive
Sasaki et al. [44]	1993	80	F	Pancytopenia	Hematological malignancy	N/A	N/A	Died
